# Supplementary material for: Spatiotemporal observation of surface plasmon polariton mediated ultrafast demagnetization
Source: Nat Commun. 2025 Jan 21;16:873. doi: 10.1038/s41467-025-56158-5 (PMC11756397; doi:10.1038/s41467-025-56158-5)
Supplement: Supplementary file 1 — Supplementary Information [file 41467_2025_56158_MOESM1_ESM.pdf]

Spatiotemporal observation of surface plasmon polariton mediated ultrafast demagnetization

Supplementary Information

Yuzhu Fan[1], Gaolong Cao[1], Sheng Jiang[2], Johan Åkerman[3], Jonas Weissenrieder[1]

[1]School of Engineering Sciences, KTH Royal Institute of Technology, Applied Physics, AlbaNova, SE-106 91 Stockholm, Sweden

[2]School of Microelectronics, South China University of Technology, 510641 Guangzhou, China;.

[3]Department of Physics, University of Gothenburg, Gothenburg, Sweden.

1 Spatial frequency of the transient magnetic grating (TMG)

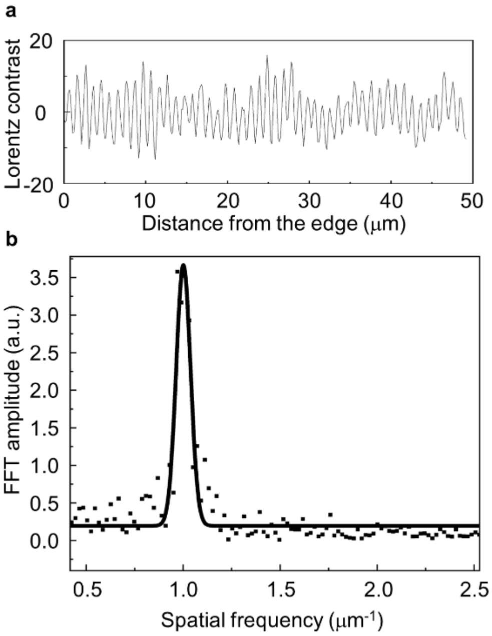

Supplementary Fig. 1 (a) Line profile with an integration width of 11 μm extracted from the raw data at 1.7 ps time delay. (b) Spatial Fast Fourier transform (FFT) analysis of the TMG contrast in (a). The spatial frequency is 1 μm, consistent with the calculated spatial frequency for the experimental geometry with a pump wavelength 1030 nm.

2 Simulation of the spatial distribution of the light intensity of the optical grating

The optical path difference between the direct pump beam and the reflected pump beam will result in a spatial dependence of the light intensity at the sample surface, with higher intensities at regions of constructive interference near the edge than at constructive regions further away from the edge. To describe the spatial distribution of the light intensity, we simulate the spatial distribution of electric field $E(t)$ of the femtosecond laser with a pulse duration of 300 fs assuming the homogeneous laser fluence. Supplementary Fig. 2 shows the simulated intensity of the interfering beams $I(t)$ as a function of relative phase shift due to the optical path difference between the direct pump beam and the reflected pump beam. An integer multiple of 2π relative phase difference represents the corresponding integer multiple of the spatial interference periodicity. By integrating the $I(t)$ of the interfering beams, we obtain the spatial distribution of the local energy of the optical grating as shown in Supplementary Fig. 3. Note that at a perpendicular distance of 40 μm from the edge, as shown in Supplementary Fig. 2 at 40 phases, the arrival time difference, due to the optical path difference, between the direct and reflected beams is 0.07 ps.

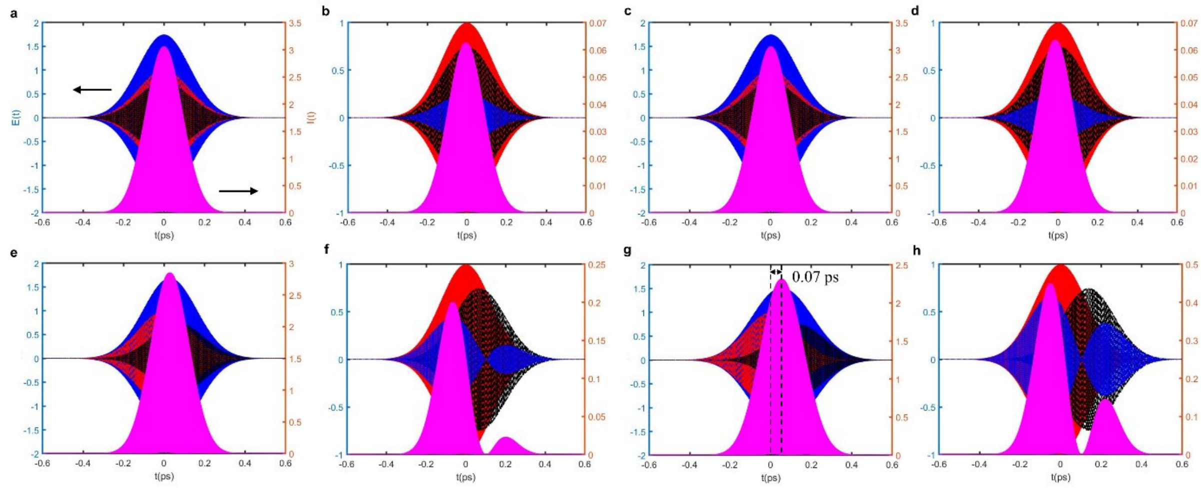

Supplementary Fig. 2 Simulated electric field $E(t)$ and intensity $I(t)$ of the interfering beams as a dependence on phase shift. The red curves represent $E(t)$ of the direct beam, the black curves represent $E(t)$ of the reflected beam with measured 50% reflectivity from the slanted permalloy surface, the blue curves represent $E(t)$ of the interference of the two beams, and the purple curves represent the intensity $I(t)$ of the interfering beams. (a)-(h) indicate that the phase difference between the direct beam and the reflected beam is 0, π, 2π, 3π, $20\times2\pi$, $20.5\times2\pi$, $40\times2\pi$, and $40.5\times2\pi$, respectively.

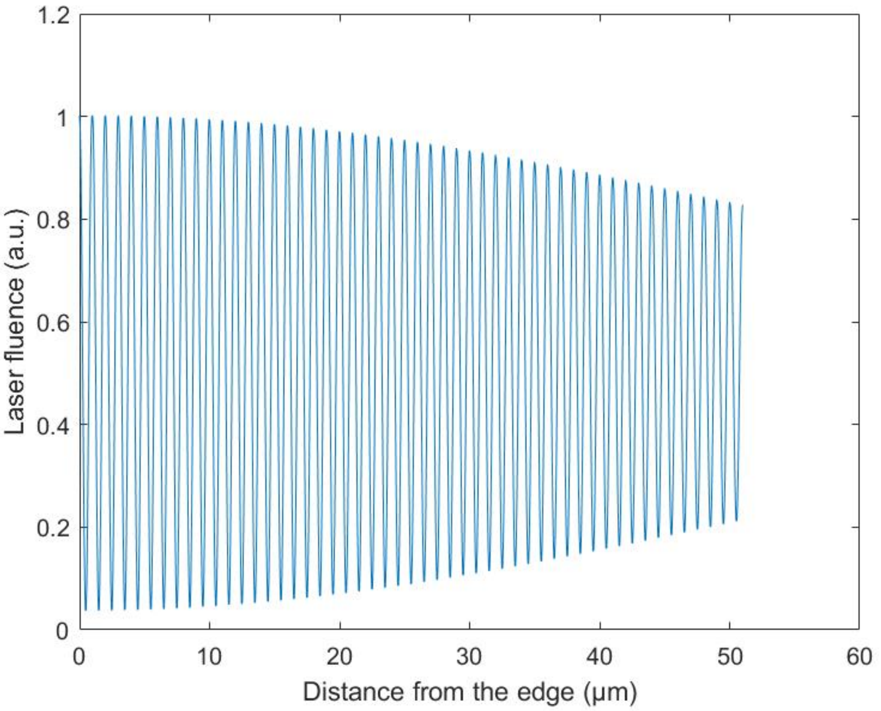

Supplementary Fig. 3 Normalized distribution of the laser fluence across the optical grating.

3 Simulation of the SPP dispersion relationship

The calculation of dispersion relationships is performed by solving Maxwell's equations in the frequency domain, using the Radio Frequency Module in the COMSOL Multiphysics® software based on the implemented Finite Element Method (FEM)[1]. The SPP is stimulated both at the top interface (Mode 1, Air/Al (5 nm)/$Ni_{80}Fe_{20}$ (50 nm)) and the bottom interface (Mode 2, $Ni_{80}Fe_{20}$ (50 nm)/ Si_3N_4 (50 nm)/Air), as shown in Supplementary Fig. 4a-c, respectively. We use the optical properties of all the involved materials from the software's built-in material library. The calculated electric field of SPP at both interfaces propagates tangentially to the interface and decays exponentially in the perpendicular direction. As expected, the decay in the perpendicular direction is faster on the metal side due to the strong absorption. Therefore, it is safe to exclude the coupling between the two SPP modes. The dispersions of free-space light and SPP after computing are shown in Supplementary Fig. 4d. At 1.2 eV (as in the case of the experiment), k_y of the free-space light is calculated as 6008639 m^{-1}, and the k_y of the SPP at the top interface is calculated as 6382473 m^{-1}. The group velocity v_g of SPP can be obtained from the relationship $v_g = dE/dk_y$. Supplementary Fig. 4e demonstrates that the incident light is almost fully absorbed after passing through the $Ni_{80}Fe_{20}$ thin film with 50 nm's thickness according to the Beer-Lambert Law. Therefore, the SPP excited by the incident light at the bottom interface is supposed to be too weak to consider. Supplementary Fig. 4f shows the simulated spatial distribution of relative SPP intensity under experimental conditions. The SPP propagation length (i.e., the distance at which the SPP intensity decreases to 1/e of its initial value) is fitted using an exponential decay function, $y = A_1 \times \exp(-x/t_1) + y_0$, where the fitted parameter t_1

represents the SPP propagation length. The fitted value for the simulated SPP propagation length is ~14 μm, consistent with the measured SPP propagation length shown in Fig. 3(c).

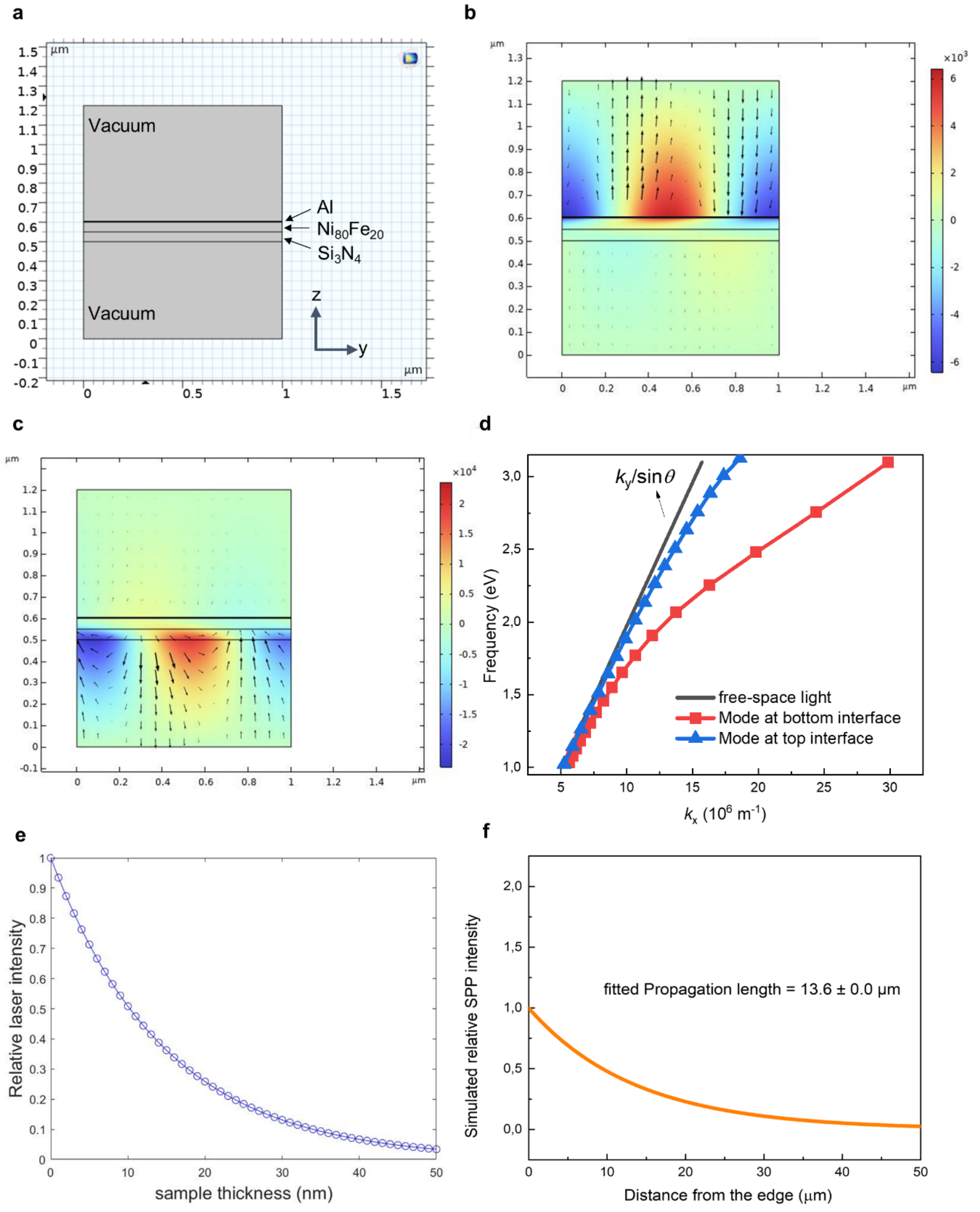

Supplementary Fig. 4 (a) Geometry of the simulation. A multilayer structure was placed in vacuum according to the experimental condition of vacuum/ Al (5 nm)/$Ni_{80}Fe_{20}$ (50 nm)/Si_3N_4 (50 nm)/vacuum. (b) Simulated y-component of the electric field distribution (V/m) of the SPP at the top interface (Mode 1) at 1.2 eV, which is abbreviated as Air/$Ni_{80}Fe_{20}$ (50nm). (c) Simulated y-component electric field distribution (V/m) of SPP at the bottom interface (Mode 2) at 1.2 eV, abbreviated as $Ni_{80}Fe_{20}$ (50 nm)/ Si_3N_4 (50 nm). (d) A plot of the simulated frequency-wave vector dispersions of the SPP at the top (blue)

and bottom (red) interfaces. The black trace represents the free-space light dispersion with an incidence angle θ of 73°. (e) Absorption of incident light as a function of thickness calculated for a $Ni_{80}Fe_{20}$ thin film according to the Beer-Lambert Law. The relevant sample thickness is 50 nm. (f) The simulated spatial distribution of relative SPP intensity over a distance of 50 μm. By applying the exponential decay fit to the data. the simulated SPP propagation length is ~14 μm, consistent with the measured SPP propagation length from Fig. 3(c).

4 Fitting results of the demagnetization time constant

The demagnetization curves in Fig. 4(b) are fitted by the following bi-exponential function[2-4]:

$$F(t) = \begin{cases} y_0 \ (t < 0) \\ y_1 + (A_1 \exp(-t/t_{demag}) + A_2 \exp(-t/t_{remag})) \otimes G(t) \ (t \geq 0) \end{cases} \quad (1)$$

where $G(t)$ is a Gaussian function describing the photoelectron (probe) pulse, A_1 and A_2 are parameters for the fit. The demagnetization time constant t_{demag}, recovery time constant t_{remag}, and fitting parameters as the dependence on the distance from the edge are exhibited in Supplementary Fig. 5. The demagnetization time constant t_{demag} represents the time required for the quench in magnetization to reach 63% of its maximum. The pulse duration of $G(t)$ (1.2 ps) used for fitting is obtained from PINEM result by using the same conditions with the demagnetization experiments.

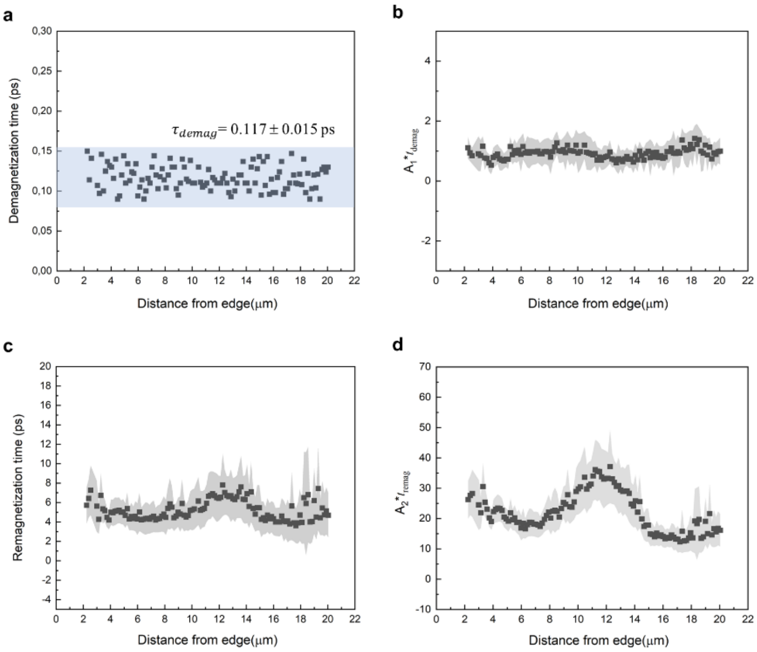

Supplementary Fig. 5 Fitting results for the (a) demagnetization time constant t_{demag}, (b) $A_1 \times t_{demag}$, (c) recovery time constant t_{remag}, and (d) $A_2 \times t_{remag}$ as a function of the distance from the edge.

5 Simulation of space-time contour of the FFT amplitude at homogeneous light absorption

The demagnetization dynamics as a function of light fluence was calculated by solving the stochastic Landau–Lifshitz–Gilbert (LLG) equation through the Heun method as implemented in the Vampire software package[5,6]. Supplementary Fig. 6a exhibits the simulated spatial distribution of the local laser fluence of the optical TG according to the distribution from Supplementary Fig. 3 with an arbitrarily assigned value of 45 mJ/cm^2 at 0 μm distance from the edge. Supplementary Fig. 6b shows simulated results for the demagnetization dynamics as a function of increasing laser fluence. The magnitude of demagnetization exhibits an approximately linear dependence with increasing laser fluence within the simulated fluence range. From the spatial distribution of the laser intensity in the optical TG simulated in Supplementary Fig. 6a, we can convert the simulated demagnetization dynamics as a function of localized laser intensity into demagnetization dynamics as a function of the distance from the edge. Supplementary Fig. 6c shows the simulated demagnetization curves as a function of spatial position by using the fluences from Supplementary Fig. 6a. In the simulations we arbitrarily assign a pump fluence of 45 mJ/cm^2 at 0 μm distance from the edge. The arbitrary choice of the initial pump fluence value at 0 μm within the simulated fluence range will not cause any difference of the simulated space-time contour of the FFT amplitude in Supplementary Fig. 6f since the demagnetization responds approximately linear with the fluence. The resulting sinusoidal distribution of magnetization can be extracted at each time delay from Supplementary Fig. 6c and a corresponding simulated LUTEM image can be generated (shown in Supplementary Fig. 6e). By using the same spatial FFT processing method as for Fig. 4(a), the simulated space-time contour of FFT amplitude is obtained, as shown in Supplementary Fig. 6f.

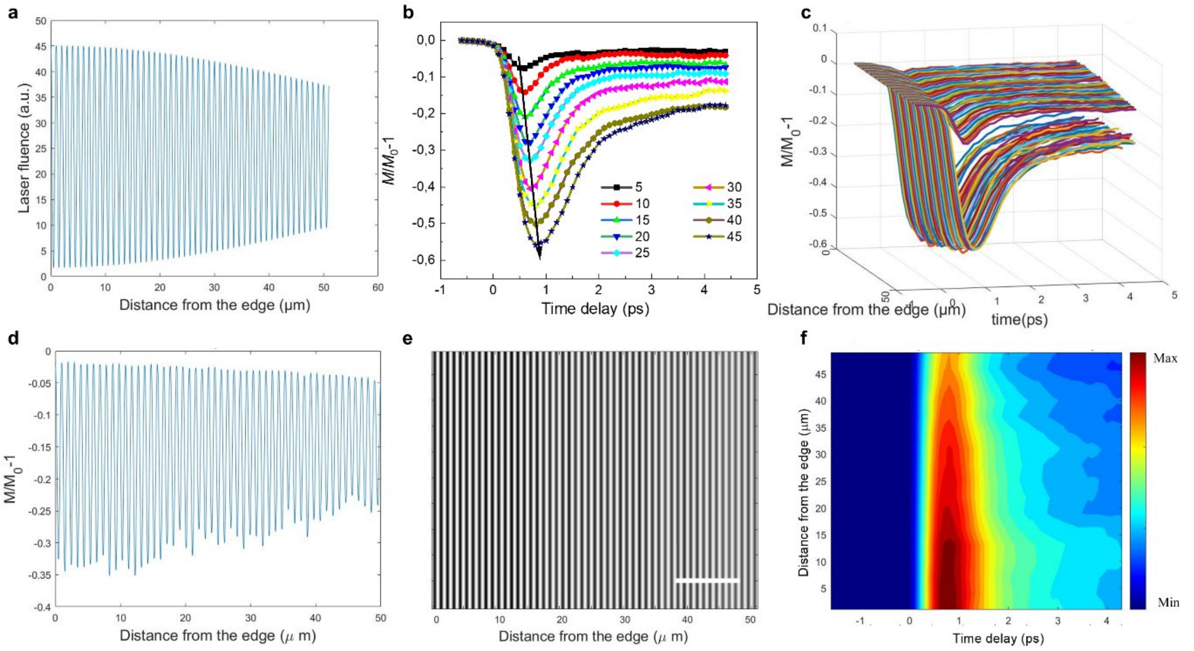

Supplementary Fig. 6 (a) Simulated spatial distribution of the local laser fluence of the optical TG (according to the distribution from Fig.S3) with an arbitrarily assigned value of 45 mJ/cm^2 at 0 μm distance from the edge. (b) Simulated demagnetization dynamics as a function of pump fluence using the Vampire software package. (c) Simulated demagnetization curves at different spatial positions using

the pump fluences from (a). (d) Simulated sinusoidal magnetization distribution (magnetic gratings) at 2 ps time delay (extracted from (c)). (e) Simulated LUTEM image at 2 ps time delay with the magnetization distribution in (d). (f) Simulated space-time contour of FFT intensity from the simulated LUTEM images (as (e)) by using the same spatial FFT processing method for Fig.4(a).

6 Control experiment without a plasmonic hotspot

To provide direct evidence of the SPP induced effects on the ultrafast demagnetization dynamics, we conducted control experiments that effectively quench the SPP field. The sample edge serves as a plasmonic hotspot for effective excitation of SPP (Supplementary Fig. 7a). By separating the mirror from the sample (Supplementary Fig. 7b), by approximately 50 μm, we eliminate the plasmonic hotspot. This geometry allows investigation of the demagnetization dynamics in absence of SPP excitation and the optical absorption of the sample can be considered spatially uniform. Thus, a comparison of the results from the geometries shown in Supplementary Fig. 7 allow us to isolate the effects from SPP excitation and study contributions to the demagnetization dynamics exclusively from the optical TG.

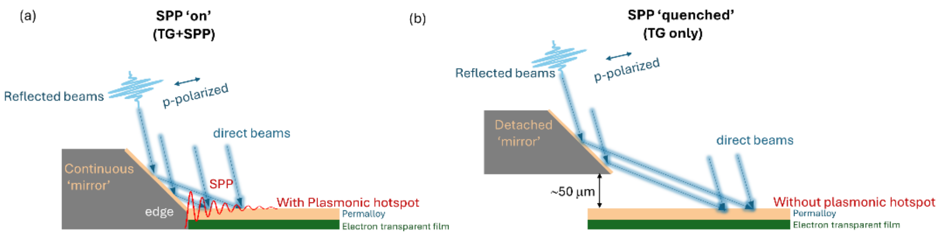

Supplementary Fig. 7 Schematic illustration of the experimental setup for (a) with a plasmonic hotspot (edge) and (b) without a plasmonic hotspot. The distance (~50 μm) between the detached mirror and the sample in (b) is measured by scanning electron microscopy (SEM).

7 Spatial time delay for reaching maximum demagnetization

Supplementary Fig. 8 presents inverse Fast Fourier Transform (FFT) filtered LUTEM results for temporal delays spanning from -0.4 ps to 1.0 ps. The primary objective of the FFT image processing is to enhance the magnetic signatures. In instances where the raw LUTEM image lacks grating signals, the filtered image manifests solely as noise, exemplified by the images corresponding to -0.4 ps in Supplementary Fig. 8. Conversely, when the raw data captures grating signals, discernible fringes emerge in the filtered image, as depicted in the image corresponding to 1 ps in Supplementary Fig. 8. Significantly, our observations reveal the initial presence of grating solely near the edge at 0 ps, as denoted by a white dashed box. Over time, the region where gratings materialize expands from the edge toward the inner regions of the sample, culminating in the entire field of view exhibiting gratings. This progression occurs over a span of approximately 0.3 ps across a distance of 50 μm from the edge, as observed in

the 0 ps to 0.3 ps panels in Supplementary Fig. 8, consistent with the tilted front shown in Fig. 4(c).

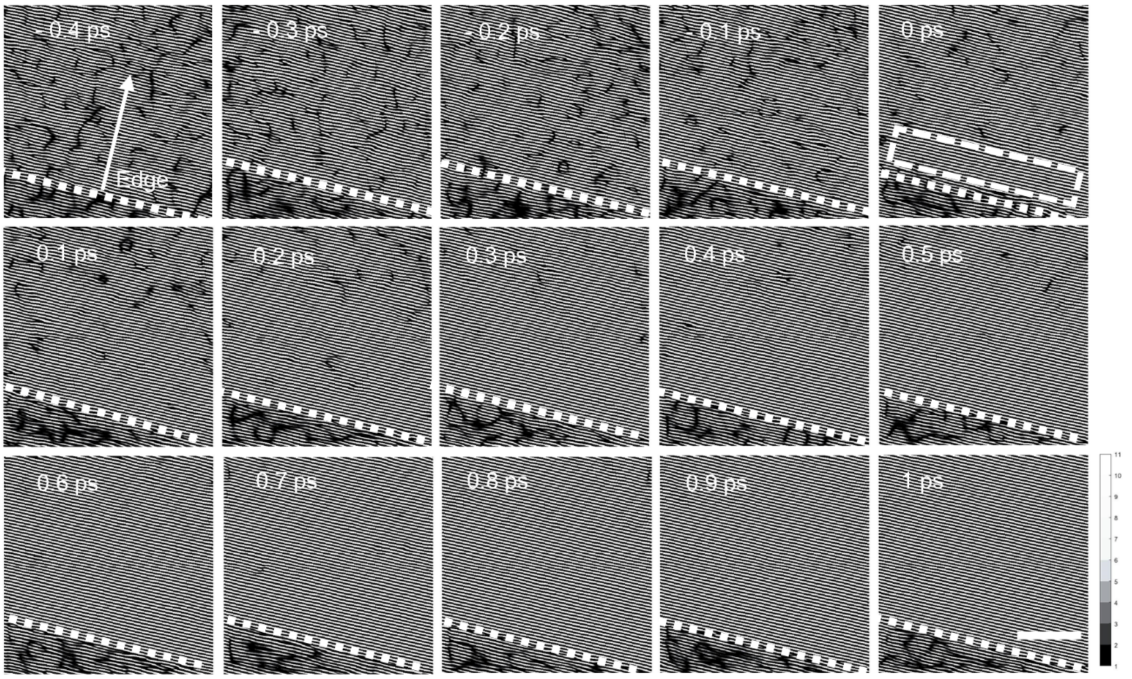

Supplementary Fig. 8 FFT Filtered Lorentz images generated through an inverse FFT process selecting the FFT spatial frequency corresponding to the TG from -0.4 ps to 1.0 ps. Formation of magnetic gratings is initially observed near the edge and will extend with time towards the inner regions of the sample. The formation of the grating at the edge occurs approximately 0.3 ps faster than at the position 50 μm away from the edge. The scale bar represents 20 μm.

Supplementary Fig. 9 shows the spatial distribution of the maximum demagnetization time in the control experiment without a plasmonic hotspot, as indicated by the purple data. The results demonstrate that the maximum demagnetization time occurs approximately 0.2 ps earlier at the edge compared to the region 50 μm away from the edge. For comparison, the orange data represents the spatial distribution of the maximum demagnetization time with an experimental geometry including a plasmonic hotspot. We fitted the slope values for both cases and found that the fitted slope for the 'without plasmonic hotspot' case is $(5.58 \pm 1.46) \times 10^{-3}$ ps/μm, while the fitted slope for the 'with plasmonic hotspot' case is $(5.83 \pm 0.74) \times 10^{-3}$ ps/μm, showing no significant difference between the two cases.

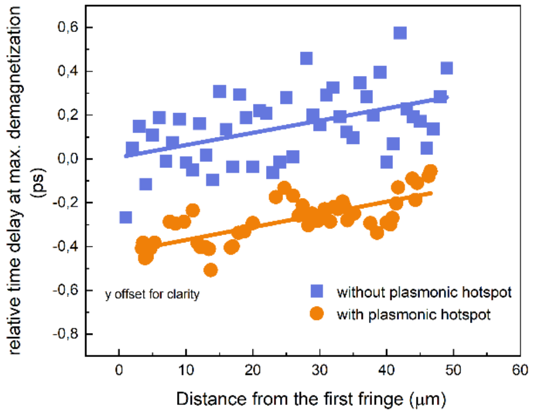

Supplementary Fig. 9 Spatial distribution of the relative time delay for reaching maximum demagnetization amplitude for experiments 'with SPP' (orange data) and 'without SPP' (purple data). The orange data has been offset along the y axis for clarity.

8 The effect of optical path difference in optical TG on maximum demagnetization time

Here, we considered the impact of the optical path difference between the reflected and direct incident beams on the relative time delay of the interfered pulse center and pulse broadening, and how these factors ultimately affect the spatial distribution of the maximum demagnetization time. The interference between the reflected and incident beams generates a transient grating, and also causes the pulse center and pulse width of the interfered light to delay and broaden, respectively, as the optical path difference increases. In Supplementary Fig. 10a and b, we calculated the relative time delay of the interfered pulse center and the pulse FWHM as a function of the optical path difference. The results indicate that for a 50 μm optical path difference, the interfered pulse center is delayed by ~0.1 ps, and the pulse FWHM is broadened by ~0.04 ps. Since the time delay in the pulse center leads to a corresponding time delay in the onset of demagnetization, the ~0.1 ps delay in the interfered pulse center results in a ~0.1 ps delay in the maximum demagnetization time at a distance of 50 μm from the edge. To investigate the effect of pulse FWHM broadening on demagnetization time, we simulated in Supplementary Fig. 10c how the broadening of the excitation pulse affects the demagnetization curves. The relationship between the extracted maximum demagnetization time and the excitation pulse broadening is shown in Supplementary Fig. 10d, with the calculations indicating that a 0.04 ps increase in pulse FWHM leads to a ~0.1 ps delay in the maximum demagnetization time. Therefore, considering only the effects of the optical path difference in the transient grating, the maximum demagnetization time is theoretically delayed by a total of 0.2 ps at 50 μm from the edge, which is consistent with the spatial distribution of maximum demagnetization time observed in the control experiment data shown in Supplementary Fig. 9. This suggests that the spatial delay in the maximum demagnetization time observed in Fig. 4b-c, Supplementary Fig. 8 and orange data in Supplementary Fig. 9 is primarily due to the optical path difference from the optical TG.

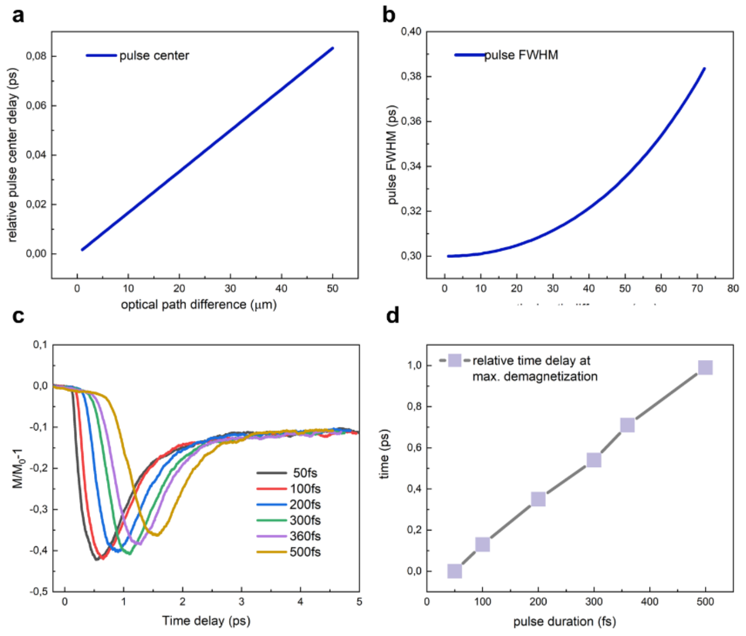

Supplementary Fig. 10 (a) The change in relative time delay at the interfered pulse center with the optical path difference; (b) The change of the FWHM of the interfered pulse with the optical path difference; (c) Demagnetization curves excited with increasing pulse FWHM, simulated by using the atomistic spin dynamics (ASD) model. Note that the centers of all excitation pulses have been aligned at same time delay; (d) The relative delay at maximum demagnetization time as a function of excitation pulse FWHM.

9 Simulation of the spatial distribution of maximum demagnetization amplitude by using the 'effective pump fluence'

The SPP intensity distribution from Fig. 3(c) and the maximum demagnetization magnitude ΔF_{max} distribution from Fig. 4(d) were combined into a single figure to illustrate their correlation, as shown in Supplementary Fig. 11a. The spatial distribution of maximum demagnetization amplitude is simulated according to the 'effective pump fluence' scaled from the spatial map of SPP intensity. The results are shown in the purple data of Supplementary Fig. 11d, where the edge shows an approximately two-fold enhancement of the maximum demagnetization amplitude. A decay constant of 20 ± 4 μm is obtained from the exponential fit. Both features are consistent with the experimental results of the spatial distribution of maximum demagnetization amplitude ΔF_{max} exhibited in the same figure in Supplementary Fig. 11d (orange data), where the edge shows an approximately two-fold enhancement in demagnetization and the decay constant is 23 ± 10 μm (from the exponential fit). All these features demonstrate that the demagnetization dynamics near the plasmonic hotspot is dominated by SPP excitation.

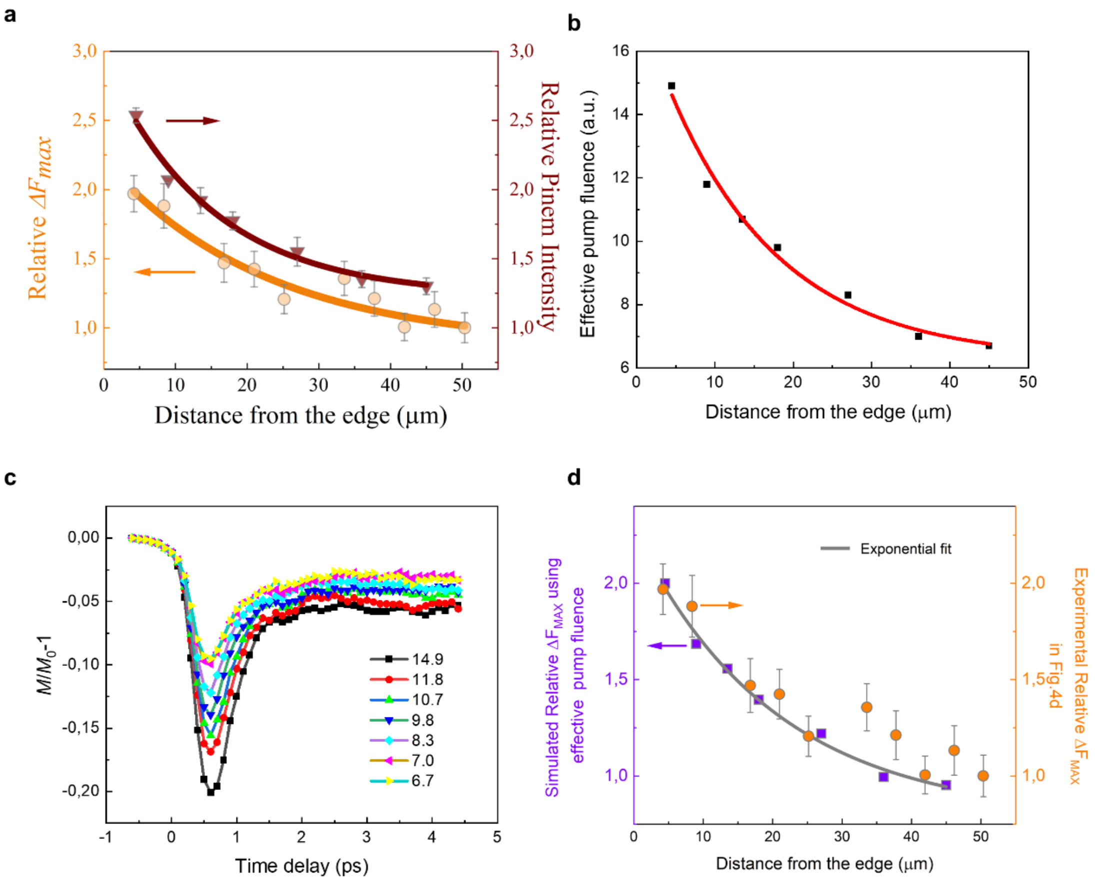

Supplementary Fig. 11 (a) The SPP intensity distribution measured from Fig. 3(c) (wine data) and the maximum demagnetization magnitude ΔF_{max} distribution from Fig. 4(d) (orange data) were combined into a single figure to illustrate their correlation. The SPP intensity distribution (wine data) has an y axis offset of 0.3 for improved visualization. (b) The spatial distribution of the 'effective pump fluence' scaled from the spatial map of SPP intensity in (a). The red line is the exponential fit. (c) Simulated demagnetization curves as a function of effective pump fluences according to the distribution in (b). (d) ΔF_{max} extracted from (c) (purple) as a function of the distance from the edge. Experimental data from Fig.4(d) is also shown here to show the correlation. The grey line is exponential fit. Since PINEM is only sensitive to SPP intensity, two datapoints in ΔF_{max} distribution at 13 and 30 μm corresponding to the beat has been removed here for improved visualization.

10 Simulation of the optical beat

The interference by the co-propagation of the reflected pump and the SPP at the top interface due to the k_y mismatch, as shown in the schematic drawing in Supplementary Fig. 12a, results in an optical beat periodicity $\lambda_{\mathrm{beat}} = 2\pi/\Delta k_y$, that is calculated as 16.81 μm as shown in Supplementary Fig. 12b. The optical beat periodicity is consistent with the experimental observation of a magnetic periodicity of ~17 μm.

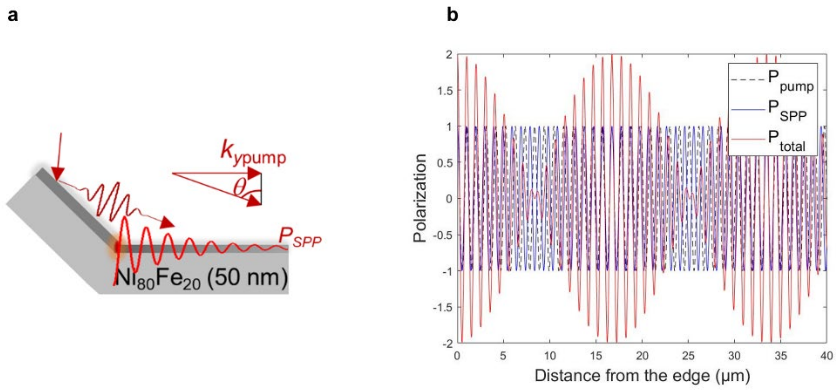

Supplementary Fig. 12 (a) Illustration of the co-propagation of the reflected pump beam and the SPP wave. (b) The dashed black line represents the polarization of the reflected pump $P_{\text{pump}}=\varepsilon_0\chi E_{\text{pump}}$, the wave vector k_{ypump} is extracted from the free light dispersion at 1.2 eV in Supplementary Fig. 4d. The blue line represents the transverse component of the SPP wave from Mode 1, the wave vector k_{ySPP} is extracted from the SPP dispersion at top interface at 1.2 eV in Supplementary Fig. 4d. The red line represents the modulation of the P_{total} by the interference between P_{pump} and P_{SPP} with a resulting beat periodicity $\lambda_{\text{beat}} = 2\pi/\Delta k_{\text{y}}$. Damping of the amplitude is not included in the simulations.

Reference

1 COMSOL AB, Stockholm, Sweden. COMSOL Multiphysics R. Version 6.1. URL: http://www.comsol.com.

2 Radu, I. *et al.* Ultrafast and Distinct Spin Dynamics in Magnetic Alloys. *Spin* **05** (2015).

3 You, W. *et al.* Revealing the Nature of the Ultrafast Magnetic Phase Transition in Ni by Correlating Extreme Ultraviolet Magneto-Optic and Photoemission Spectroscopies. *Phys Rev Lett* **121**, 077204 (2018).

4 Tengdin, P. *et al.* Critical behavior within 20 fs drives the out-of-equilibrium laser-induced magnetic phase transition in nickel. *Sci Adv* **4**, eaap9744 (2018).

5 Evans, R. F. *et al.* Atomistic spin model simulations of magnetic nanomaterials. *J Phys Condens Matter* **26**, 103202 (2014).

6 VAMPIRE software package, version 6.0 Available from https://vampire.york.ac.uk/.
